# Impact of passive heat stress and passive heat acclimation on circulating extracellular vesicles: An exploratory analysis

**DOI:** 10.1113/EP090823

**Published:** 2023-01-09

**Authors:** Nicholas Ravanelli, Hadiatou Barry, Anthony R. Bain, Laurent Vachon, Catherine Martel, Daniel Gagnon

**Affiliations:** ^1^ School of Kinesiology Lakehead University Thunder Bay Ontario Canada; ^2^ Montreal Heart Institute Montreal Quebec Canada; ^3^ Department of Pharmacology and Physiology Université de Montréal Montreal Quebec Canada; ^4^ Department of Kinesiology University of Windsor Windsor Ontario Canada; ^5^ Department of Medicine Université de Montréal Montreal Quebec Canada; ^6^ School of Kinesiology and Exercise Science Université de Montréal Montreal Quebec Canada

**Keywords:** extracellular vesicles, heat, hyperthermia, plasma, temperature

## Abstract

This retrospective exploratory analysis aimed to improve our understanding of the effect of passive heat stress and subsequent heat acclimation on the circulating concentration of extracellular vesicles (EVs). Healthy young adults (four females and six males, 25 ± 4 years of age, 1.72 ± 0.08 m in height and weighing 71.6 ± 9.0 kg) were heated with a water‐perfused suit before and after seven consecutive days of hot water immersion. Pre‐acclimation, participants were heated until oesophageal temperature increased to ∼1.4°C above baseline values. Post‐acclimation, participants were heated until oesophageal temperature reached the same absolute value as the pre‐acclimation visit (∼38.2°C). Venous blood samples were obtained before and at the end of passive heating to quantify plasma concentrations of EVs from all cell types (CSFE^+^), all cell types except erythrocytes (CSFE^+^MHCI^+^), platelets (CSFE^+^MHCI^+^CD41^+^), endothelial cells (CSFE^+^MHCI^+^CD62e^+^), red blood cells (CSFE^+^CD235a^+^) and leucocytes (CSFE^+^MHCI^+^CD45^+^) via flow cytometry. Passive heat stress increased the concentration of CFSE^+^ EVs (46,150,000/ml [3,620,784, 88,679,216], *P* = 0.036), CFSE^+^MHCI^+^ EVs (28,787,500/ml [9,851,127, 47,723,873], *P* = 0.021) and CSFE^+^MHCI^+^CD41^+^ EVs (28,343,500/ml [9,637,432, 47,049,568], *P* = 0.008). The concentration of CSFE^+^MHCI^+^CD62e^+^ EVs (94,230/ml [−55,099, 243,559], *P* = 0.187), CSFE^+^CD235a^+^ EVs (−1,414/ml [−15,709, 12,882], *P* = 0.403) or CSFE^+^MHCI^+^CD45^+^ EVs (−192,915/ml [−690,166, 304,336], *P* = 0.828) did not differ during heat stress. The change in circulating EVs during passive heat stress did not differ after heat acclimation (thermal state × acclimation interactions, all *P* ≥ 0.180). These results demonstrate that passive heat stress increases the circulating concentration of total and platelet EVs and that passive heat acclimation does not alter this increase.

## Abstract (highlights)

- **What is the central question of this study?**
  How does passive heat stress and subsequent heat acclimation affect the circulating concentration of extracellular vesicles?
- **What is the main finding and its importance?**
  Passive heat stress increased the circulating concentration of total and platelet extracellular vesicles. Seven days of hot water immersion did not modify the change in circulating concentrations of extracellular vesicles during passive heat stress.

## 1 INTRODUCTION

The cellular responses to heat stress progress along a continuum, from improved cellular thermotolerance following repeated moderate heat exposure to cellular apoptosis during severe heat strain. These cellular outcomes are mediated by intra‐ and extracellular signals, including alarmins, endotoxins, microRNAs and chromatin modifiers (Bouchama et al., 2022; Horowitz, 2014; Murray et al., 2022). Accumulating, albeit still limited, evidence suggests that extracellular vesicles (EVs) might also contribute to cellular signalling and therefore the cellular adaptations in response to heat stress (Bain et al., 2017; Bouchama et al., 2008; Coombs et al., 2019; Wilhelm et al., 2017).

Extracellular vesicles are secreted into the circulation by cells in response to physical or chemical stress, during cell death and apoptosis, but also by healthy cells for intercellular communication (Shah et al., 2018; Tkach & Théry, 2016). All studied cell types secrete EVs that express markers and carry cargo specific to their cell of origin (Shah et al., 2018). Importantly, EVs contribute to systemic and organ‐specific physiological and pathophysiological processes (Shah et al., 2018). In the context of heat stress, a role for EVs was first identified by Bouchama et al. (2008). In a non‐human primate model of severe heatstroke, an increase in platelet EVs was observed >45 h post‐heat stroke, and a large proportion of these EVs contained functionally active tissue factor, suggesting that they might contribute to the excessive activation of coagulation during heatstroke. In an effort to translate these findings to humans, Bain et al. (2017) first explored the effect of heat stress on circulating concentrations of platelet and endothelial EVs. In contrast to their hypothesis, a marked reduction in endothelial and platelet EVs was observed when young healthy adults were passively heated until core temperature increased by ∼2°C. In contrast, Wilhelm et al. (2017) subsequently observed a general increase in platelet EVs during mild (∼0.5°C core temperature increase) and moderate (∼1.1°C increase in core temperature) hyperthermia in young healthy adults, but no change in endothelial EVs. Lastly, Coombs et al. (2019) did not observe a change in concentrations of endothelial, platelet, monocyte or leucocyte EVs during mild hyperthermia (∼0.35°C increase in core temperature) in healthy adults. However, a reduction in endothelial EVs was observed in a subset (*n* = 6) of unmedicated individuals with spinal cord injury who displayed greater basal concentrations relative to healthy adults.

Taken together, the effect of heat stress on circulating EVs remains unclear in humans because some studies found a decrease in platelet and/or endothelial EVs, whereas another found an increase in platelet EVs with no change in endothelial EVs. These divergent results might stem from differences in the strategy to identify EVs, the degree of hyperthermia attained and/or the clinical status of the participants. It is also not known whether heat acclimation can alter basal concentrations of circulating EVs and/or their change during acute heat stress. We recently reported on physiological adaptations in response to short‐term controlled hyperthermia heat acclimation (Barry et al., 2020, 2022; Gendron et al., 2021; Ravanelli et al., 2021; Trachsel et al., 2020). As part of this study, blood samples were obtained before and during passive heat stress, both pre‐ and post‐acclimation. This provided us with the opportunity to explore the effect of heat stress and subsequent heat acclimation on circulating EVs.

## 2 METHODS

### 2.1 Ethical approval

The study was approved by the Research Ethics and New Technologies Development Committee of the Montreal Heart Institute (#2016‐2083). All participants provided verbal and written informed consent. The study conformed to the standard set by the *Declaration of Helsinki*, except for registration in a database.

### 2.2 Participants

The data presented in this manuscript were collected as part of a larger study that was designed to determine the impact of heat acclimation on the neural control of body temperature (Barry et al., 2020). A total of 14 participants completed the study, but blood samples to measure circulating EV concentrations were available from only 10 participants (four females and six males, 25 ± 4 years of age, 1.72 ± 0.08 m tall and weighig 71.6 ± 9.0 kg, with a physical activity level of 156 ± 71 min/week). Considering the retrospective and exploratory nature of the present analyses, we did not formulate an a priori hypothesis nor did we perform an a priori power calculation.

### 2.3 Study design

Participants completed two experimental visits, one before and one after a 7‐day passive heat acclimation protocol. Before all visits, participants were instructed to consume ∼500 ml of water and eat a light snack in the morning and to avoid coffee, alcohol and strenuous exercise for 12 h. Hydration status was assessed upon arrival for each laboratory visit. If urine specific gravity was >1.025, participants were asked to drink 250 ml of water before continuing with the visit; this occurred once during the post‐acclimation visit. Urine specific gravity did not differ between the pre‐acclimation (1.015 ± 0.008) and post‐acclimation (1.012 ± 0.007) visits (*P* = 0.47). For female participants, the first experimental visit occurred between days 9 and 17 of the menstrual cycle. Two female participants were taking oral contraceptives.

### 2.4 Experimental visits

All visits started in the morning (08.00–09.00 h). Participants arrived in the laboratory, voided their bladder, and weighed themselves nude. Afterwards, participants were instrumented and wore a water‐perfused suit (COOLTUBEsuit; Med‐Eng) before resting supine while 29−30°C water (AD07H200; PolyScience) was circulated through the suit for a minimum of 10 min, at the end of which two venous blood samples were withdrawn. The temperature of the water perfusing the suit was subsequently increased to 50°C and maintained at this temperature until oesophageal temperature increased ≥1.2°C above baseline values. This increase in core temperature was targeted to induce a robust increase in skin sympathetic nerve activity to test the primary hypothesis of the study. During the post‐acclimation visit, heat exposure continued until oesophageal temperature reached the same absolute temperature attained pre‐acclimation. Blood samples were obtained within the last 5 min of heating, after which the water perfusing the suit was reduced to cool the participant. Participants were subsequently de‐instrumented before weighing themselves nude.

### 2.5 Heat acclimation protocol

Heat acclimation involved 7 consecutive days of controlled hyperthermia. Participants were initially immersed to shoulder level in a circulated water bath maintained at 40–41°C until rectal temperature reached 38.6°C (∼30 min). Participants then sat on a bench placed within the bath so that the water was at waist level to maintain rectal temperature ≥38.6°C for an additional 60 min. The pre‐acclimation visit was performed within 7 days of the first acclimation visit. The post‐acclimation visit was performed within 12–48 h of the last acclimation visit.

### 2.6 Measurements

Urine specific gravity was measured using a digital refractometer (PAL‐10S; Atago). Oesophageal temperature was measured using a paediatric grade thermistor (400 series, 9Fr; Covidien). Mean skin temperature was calculated as a weighted average of measurements on the arm, chest, thigh and calf (Ramanathan, 1964) using t‐type thermocouple wire (EXPP‐T‐20‐SLE‐500) connected to a thermocouple reader (TC‐2000; Sable Systems International). Heart rate was calculated from the R–R interval of a five‐lead ECG signal (Solar i8000; GE Healthcare). Continuous data were recorded at 1,000 Hz with a data acquisition system (PowerLab 16/35; AD Instruments). Systolic and diastolic blood pressures were measured using an automated monitor (Tango M2; SunTech Medical) to calculate mean arterial pressure (one‐third systolic × two‐thirds diastolic). Nude body weight was measured with a digital scale (IND236; Mettler‐Toledo; precision, 0.01 kg) to calculate whole‐body sweat rate. Blood samples drawn in K2 EDTA vacutainers were analysed for a full blood count (DxH 800; Beckman Coulter). Changes in haemoglobin and haematocrit were used to calculate the relative change in plasma volume during passive heat stress (Dill & Costill, 1974).

### 2.7 Quantification and characterization of EVs

Blood samples were drawn into a vacutainer with sodium citrate and immediately centrifuged (1,000*g* for 15 min) to aliquot and store plasma at −80°C until analyses. Plasma samples were thawed at 37°C, diluted in FACS buffer [10 mM Hepes (pH 7.4), 140 mM NaCl and 2.5 mM CaCl_2_, filtered at 0.22 μm] and incubated for 30 min at 37°C with anti‐MHCI (1:400; BD Pharmigen; catalogue no. 555553), anti‐CD235a BV786 (1:1,000; BD Biosciences; catalogue no. 740984), anti‐CD41 PerCP Cy5.5 (1:200; BioLegend; catalogue no. 303720), anti‐CD62e BV605 (1:150; Biolegend cat. #304906) and anti‐CD45 V500 (1:200; BD Biosciences; catalogue no. 560777) antibodies. Membrane dye carboxyfluorescein diacetate succinimidyl ester (1 mM CFSE; Thermo Scientific) and 10 mM d‐Phe‐Pro‐Arg‐chloromethylketone (PPACK; Cayman Chemical) were also added to the antibody mix. Samples were analysed with a BD FACSCelesta flow cytometer (BD Biosciences) equipped with a 20 mW blue laser (488 nm), a 40 mW red laser (640 nm) and a 50 mW violet laser (405 nm) in the BVR12 configuration. The machine was calibrated before each analysis with Research Beads (BD Biosciences) to provide a standardized method to perform quality control of the optics, electronics and fluidics of the instrument. The sheath fluid [PBS (pH 7.4)] was filtered on 0.1 nm bottle top filters (Sarstedt, Newton, NC, USA) before sheath tank filling. The 450/40 bandpass filter (BV421; violet laser) was manually swapped after cytometer setup and tracking calibration with a 1‐mm‐thick magnetron sputtered 405/10 bandpass filter (Chroma Technology). Count beads (Apogee Flow System; catalogue no. 1426) were added to determine the concentration of EVs according to Equation 1:

(1) [EVs]=No.ofEVsNo.ofCBs×VolumeofCB×[initialCB]Totalvolume×DF−[EVsBg]12×1,000,

where [EVs] is the EV concentration in the sample; No. of EVs is the number of EVs of interest during analysis; No. of CBs is number of count beads during analysis; Volume of CB is the volume of count beads added to the sample; [initial CB] is the initial concentration of count beads; Total volume is the total volume of the analysed sample; DF is the dilution factor of the EV sample; and [EVs Bg] is the concentration of EVs in the background tube.

**FIGURE 1:**
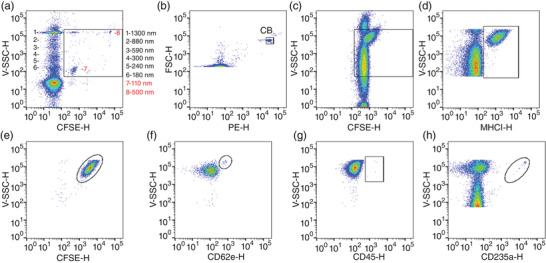
Flow cytometry gating strategy for identifying extracellular vesicles. (a) Megamix calibration beads (Apogee Flow System) ranging from 180 to 1,300 nm were used to identify events with similar size and granularity. (b) Phycoerythrin (PE) positive count beads (CB; 1 μm; Apogee Flow System) were used to calculate the concentration of events of interest. (c) CSFE, (d) MHCI, (e) CD41, (f) CD62e, (g) CD45 and (h) CD235a positive extracellular vesicles were identified.

The flow cytometry gating strategy (Figure 1) allowed for identification total plasma EVs (CFSE^+^) and total plasma EVs except red blood cell (RBC) EVs (CFSE^+^MHCI^+^), because MHC class I is found on the cell surface of all nucleated cells in the bodies of vertebrates and on platelets, but not on RBCs. The RBC EVs were identified as CFSE^+^CD235a^+^ events. To distinguish platelet, endothelial cell and leucocyte EVs from each other, CFSE^+^MHCI^+^ events were plotted against CD41, CD62e and CD45, respectively. The flow cytometer was calibrated for EV detection using the Apogee Mix (#1493; Apogee Flow Systems, Hemel Hempstead, UK), a mixture of non‐fluorescent silica beads (180, 240, 300, 590, 880 and 1,300 nm), FITC‐fluorescent latex beads (110 and 500 nm) and count beads. Events were acquired at the lowest possible flow rate (12 μl/min) to avoid swarming effects and coincident detection (Poncelet et al., 2016). Data analysis was performed with FlowJo (v.10.5; Ashland, OR, USA).

### 2.8 Statistical analysis

Two‐way repeated‐measures ANOVAs were used to analyse the dependent variables with the factors of thermal state (normothermic, hyperthermic) and heat acclimation (pre, post). Sphericity was not assumed, and a Geisser–Greenhouse correction was applied. Post‐hoc comparisons were performed with Fisher's LSD test. Whole‐body sweat rate and change in plasma volume were compared with Student's paired *t*‐test. The critical *P*‐value was set at <0.05. Descriptive results are presented as the mean ± SD. Changes are presented as the mean difference with 95% confidence interval. Effect sizes are presented as Cohen's *d*_av_, calculated with the average standard deviation of repeated measures. Analyses were conducted with Prism software (v.8.0; GraphPad, La Jolla, CA, USA).

## 3 RESULTS

Table 1 presents the physiological responses during heat stress. Baseline oesophageal temperature was lower post‐acclimation (*P* = 0.001). By design, end‐heating oesophageal temperature did not differ between visits (*P* = 0.33). Skin temperature did not differ at baseline (*P* = 0.12) or end‐heating (*P* = 0.27). Whole‐body sweat rate was greater post‐acclimation (*P* < 0.01). Baseline (*P* = 0.15) and end‐heating (*P* = 0.11) heart rate did not differ between visits. Mean arterial pressure did not differ between visits at baseline (*P* = 0.69) or end‐heating (*P* = 0.13). Baseline (*P* = 0.90) and end‐heating (*P* = 0.82) leucocyte concentrations did not differ between visits. Post‐acclimation erythrocyte concentration was lower at baseline (*P* = 0.02) but not end‐heating (*P* = 0.64). Platelet concentrations did not differ between visits at baseline (*P* = 0.97) or end‐heating (*P* = 0.37). The relative change in plasma volume did not differ between visits (*P* = 0.07).

**TABLE 1:** Physiological responses at baseline and during passive heat stress before and after heat acclimation.

	**Pre‐acclimation**			**Post‐acclimation**		
**Parameter**	**Baseline**	**Heat**	**Change**	**Baseline**	**Heat**	**Change**
Oesophageal temperature (°C)	36.9 ± 0.3	38.3 ± 0.2	1.4 ± 0.2	36.6 ± 0.4*	38.2 ± 0.3	1.6 ± 0.5*
Mean skin temperature (°C)	33.8 ± 0.6	38.6 ± 0.6	4.8 ± 0.8	33.6 ± 0.6	38.7 ± 0.6	5.2 ± 0.6
Heart rate (beats/min)	61 ± 10	103 ± 16	42 ± 10	58 ± 9	100 ± 16	43 ± 11
Mean arterial pressure (mmHg)	86 ± 7	81 ± 9	−5 ± 12	85 ± 8	82 ± 11	−3 ± 12
Sweat rate (L/h)	–	–	0.98 ± 0.43	–	–	1.79 ± 0.73*
Leucocytes (×10^9^/L)	5.8 ± 2.3	7.6 ± 2.6	1.9 ± 2.0	6.0 ± 2.5	7.4 ± 3.2	1.4 ± 1.1
Erythrocytes (×10^12^/L)	4.7 ± 0.4	4.8 ± 0.3	0.1 ± 0.3	4.4 ± 0.4*	4.7 ± 0.3	0.3 ± 0.2*
Platelets (×10^11^/L)	2.1 ± 0.5	2.7 ± 0.8	0.6 ± 0.6	2.2 ± 0.6	2.6 ± 0.8	0.4 ± 0.3
Change in plasma volume (%)	–	–	−7.9 ± 7.4	–	–	−13.1 ± 7.3

*Note*: Values are presented as the mean ± SD for *n* = 10 subjects.

* *P* < 0.05 vs. pre‐acclimation.

**FIGURE 2:**
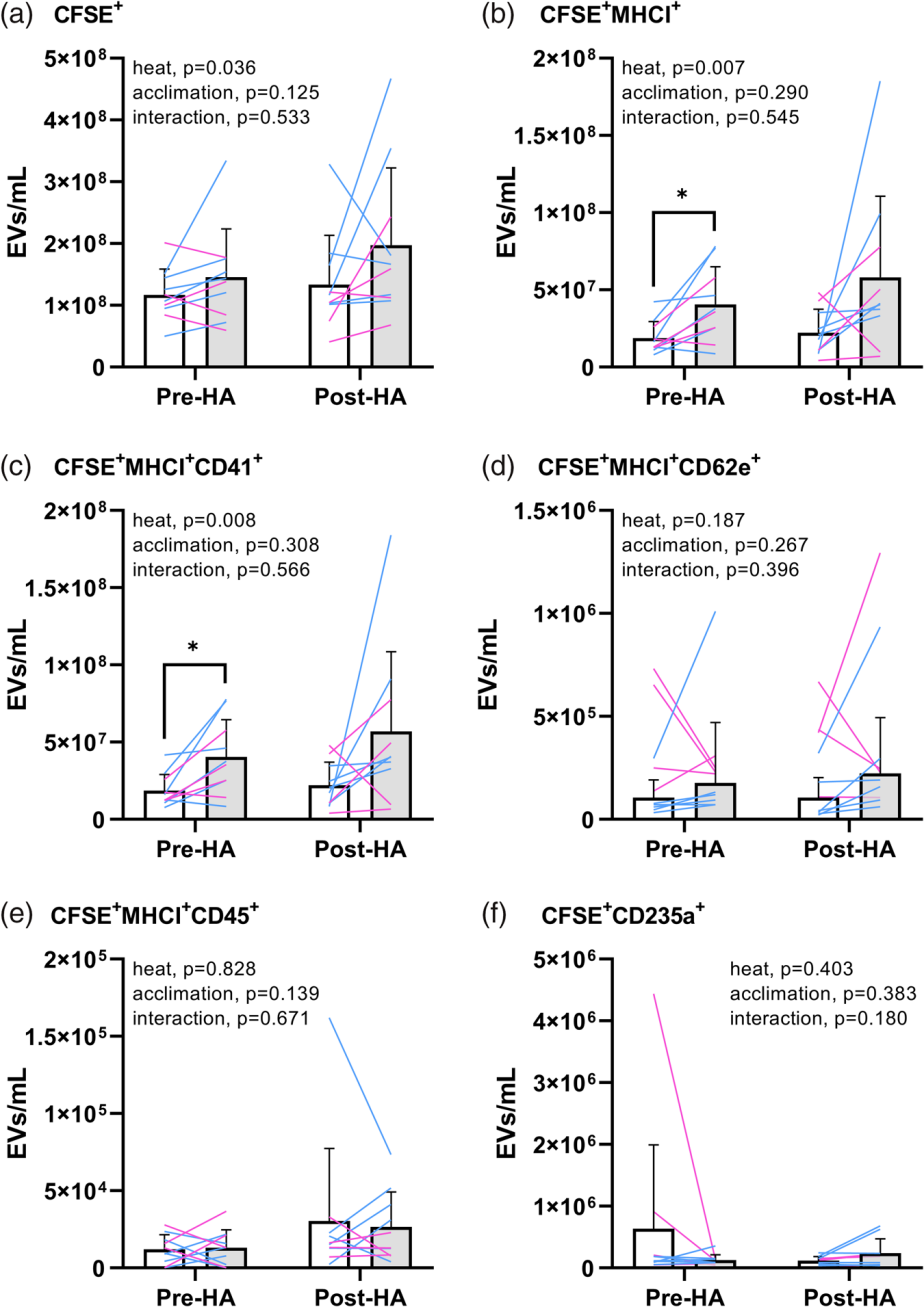
The concentration of circulating extracellular vesicles (EVs) in thermoneutral (baseline, white bars) and heat stress (grey bars) conditions before (Pre‐HA) and after (Post‐HA) 7 consecutive days of passive heat acclimation. Values are presented as the mean ± SD, with individual values for males (*n* = 6; blue lines) and females (*n* = 4; pink lines). (a) CFSE^+^, EVs from all cellular origins. (b) CFSE^+^MHCI^+^, EVs from all cellular origins except erythrocytes. (c) CFSE^+^MHCI^+^CD41^+^, EVs of platelet origin. (d) CFSE^+^MHCI^+^CD62e^+^, EVs of endothelial cell origin. (e) CFSE^+^MHCI^+^CD45^+^, EVs of leucocyte origin. (f) CFSE^+^CD235a^+^, EVs of red blood cell origin. The *P*‐values represent main effects of heat stress and heat acclimation, in addition to interaction from a two‐way repeated‐measures ANOVA. ^*^ *P* ≤ 0.05 vs. thermoneutral with Fisher's LSD post‐hoc test.

Concentrations of EVs are presented in Figure 2 and Table 2. Passive heat stress increased CFSE^+^ EVs (main effect; *P* = 0.036), CFSE^+^MHCI^+^ EVs (main effect; *P* = 0.007) and platelet EVs (main effect, *P* = 0.008). Post‐hoc comparisons revealed a significant increase in CFSE^+^MHCI^+^ EVs (*P* = 0.011 vs. thermoneutral) and platelet EVs (*P* = 0.010 vs. thermoneutral) pre‐acclimation. Endothelial EVs (main effect, *P* = 0.187), leucocyte EVs (main effect, *P* = 0.828) and RBC EVs (main effect, *P* = 0.403) did not differ during heat stress. Basal concentrations of CFSE^+^ EVs (*P* = 0.541), CFSE^+^MHCI^+^ EVs (*P* = 0.549), platelet EVs (*P* = 0.545), endothelial EVs (*P* = 0.980), leucocyte EVs (*P* = 0.206) and RBC EVs (*P* = 0.262) did not differ post‐acclimation. The change during heat stress in CFSE^+^ EVs (interaction, *P* = 0.533), CFSE^+^MHCI^+^ EVs (interaction, *P* = 0.545), platelet EVs (interaction, *P* = 0.566), endothelial EVs (interaction, *P* = 0.396), leucocyte EVs (interaction, *P* = 0.671) and RBC EVs (interaction, *P* = 0.180) did not differ post‐acclimation. The change in EV concentration during heat stress was correlated negatively with the change in plasma volume, and this correlation was significant for CFSE^+^MHCI^+^ EVs (*r* = −0.501, *P* = 0.024), platelet EVs (*r* = −0.499, *P* = 0.025) and endothelial EVs (*r* = −0.458, *P* = 0.042), but not for CFSE^+^ EVs (*r* = −0.402, *P* = 0.079), leucocyte EVs (*r* = −0.225, *P* = 0.341) or RBC EVs (*r* = −0.380, *P* = 0.099). Correcting concentrations for change in plasma volume did not alter the results, except for the increase during heat stress in CFSE^+^ EVs, which was no longer significant (main effect, *P* = 0.128).

**TABLE 2:** Extracellular vesicles during passive heat stress before and after heat acclimation.

**Extracellular vesicles (/ml)**	**Pre‐acclimation**				**Post‐acclimation**			
	**Baseline**	**Heat**	**Δ [95% CI]**	***d*_av_**	**Baseline**	**Heat**	**Δ [95% CI]**	***d*_av_**
CFSE^+^	117,060,000 ± 41,530,958	145,580,000 ± 78,085,335	28,520,000 [−15,593,751, 72,633,751]	0.48	133,510,000 ± 79,512,828	197,290,000 ± 125,055,529	63,780,000 [−32,386,290, 159,946,290]	0.62
CFSE^+^MHCI^+^	18,796,000 ± 10,631,303	40,599,000 ± 24,242,717	21,803,000 [6,463,844, 37,142,156]	1.25	22,269,000 ± 15,132,624	58,041,000 ± 52,607,568	35,772,000 [−5,974,389, 77,518,389]	1.06
CFSE^+^MHCI^+^CD41^+^	18,534,000 ± 10,603,920	40,367,000 ± 24,198,880	21,833,000 [6,567,509, 37,098,491]	1.25	22,022,000 ± 15,118,631	56,876,000 ± 51,748,743	34,854,000 [−6,272,793, 75,980,793]	1.04
CFSE^+^MHCI^+^CD62e^+^	105,120 ± 87,277	176,370 ± 293,939	71,250 [−97,769, 240,269]	0.37	105,830 ± 97,576	223,040 ± 271,443	117,210 [−33,855, 268,275]	0.64
CFSE^+^MHCI^+^CD45^+^	12,084 ± 9,452	13,166 ± 11,550	1,082 [−9,555, 11,719]	0.10	30,462 ± 46,983	26,553 ± 22,732	−3,909 [−28,938, 21,120]	0.11
CFSE^+^CD235a^+^	633,690 ± 1,361,264	126,710 ± 88,254	−506,980 [−1,482,617, 468,657]	0.70	119,210 ± 67,823	240,360 ± 231,291	121,150 [−19,872, 262,172]	0.81

*Note*: Values are presented as the mean ± SD or mean difference (Δ, heat minus baseline) with 95% confidence interval (CI) for *n* = 10 subjects.

Abbreviations: CFSE^+^, EVs from all cellular origins; CFSE^+^MHCI^+^, EVs from all cellular origins except erythrocytes; CFSE^+^MHCI^+^CD41^+^, EVs of platelet origin; CFSE^+^MHCI^+^CD62e^+^, EVs of endothelial cell origin; CFSE^+^MHCI^+^CD45^+^, EVs of leucocyte origin; CFSE^+^CD235a^+^, EVs of erythrocyte origin; *d*_av_, Cohen's *d* calculated with the average standard deviation; EVs, extracellular vesicles.

## 4 DISCUSSION

This exploratory analysis aimed to clarify the effect of passive heat stress and subsequent heat acclimation on circulating EVs. The main findings are that passive heat stress increases the concentration of total and platelet EVs and that heat acclimation does not affect this increase. Furthermore, passive heat stress does not alter the circulating concentration of endothelial, leucocyte or erythrocyte EVs, and heat acclimation does not alter basal concentrations of circulating EVs.

The effect of heat stress on circulating concentrations of EVs remains an emerging field of research. We observed an increase in the concentration of total and platelet EVs. In contrast, there were no differences in the concentration of endothelial, leucocyte and erythrocyte EVs during heat stress. These findings are in contrast to the initial observations of Bain et al. (2017) that passive heat stress decreases endothelial and platelet EVs, but in agreement with the results of Wilhelm et al. (2017) that heat stress increases platelet EVs without affecting endothelial EVs. The present results are also consistent with those reported by Coombs et al. (2019) that endothelial and leucocyte EVs do not change during heat stress in healthy adults. An explanation for the divergent responses observed by Bain et al. (2017) relative to subsequent studies remains speculative, but the level of hyperthermia and/or the strategy used to identify EVs might contribute. Regarding the level of hyperthermia, the decrease in endothelial and platelet EVs observed by Bain et al. (2017) occurred at an increase in core temperature of ∼2°C, whereas the increase in platelet EVs (and unchanged endothelial EVs) observed by Wilhelm et al. (2017) and the present analysis occurred at an increase in core temperature of ∼1.2–1.6°C. The appearance of EVs in the circulation might be modulated by the magnitude of thermal strain. Wilhelm et al. (2017) provided some evidence for this possibility, in that a more marked increase in platelet EVs was observed at an increase in core temperature of ∼1.2°C relative to an increase of ∼0.5°C. A temperature‐dependent EV response could be mediated, amongst other factors, by the respiratory alkalosis that accompanies severe hyperthermia. In vitro, the release of EVs is pH dependent, whereby concentrations are greater in acidic environments but reduced with alkalosis (Nakase et al., 2021). Future studies are needed to determine thermal strain‐dependent changes in circulating EVs during heat stress and a possible contribution of respiratory alkalosis. Regarding the strategy adopted to identify EVs, isolating and characterizing EVs is challenging, and variable approaches have been used (Nieuwland et al., 2022). When contrasting the observations made by Bain et al. (2017) to those of Wilhelm et al. (2017) and the present analysis, it should be noted that Bain et al. (2017) identified platelet EVs using P‐selectin (CD62P), whereas Wilhelm et al. (2017) and the present analysis used CD41. P‐Selectin is expressed on platelets, but also on megakaryocytes and endothelial cells, whereas CD41 is expressed on platelets and megakaryocytes. It should also be noted that EVs were quantified from arterial blood by Bain et al. (2017), whereas we used venous blood. Wilhelm et al. (2017) quantified circulating EVs in the radial artery and femoral vein during passive heat stress. During mild heat strain (core temperature increase of ∼0.5°C), platelet EVs from arterial blood remained unchanged, whereas a tendency for an increase was observed in venous blood. During more moderate heat strain (core temperature increase of ∼1.2°C), an increase in arterial but not venous EVs was observed.

An increase in circulating platelet EVs is indicative of platelet activation; therefore, platelets EVs have been implicated in haemostasis and inflammation (Puhm et al., 2021). However, ascribing a functional role to platelet EVs is not straightforward, because they can exert both pro‐ and anticoagulant or inflammatory activity depending upon the subtype of EV released and the agonist (Puhm et al., 2021). Accordingly, the functional significance of the observed increase in platelet EVs during heat stress remains uncertain. An increase in platelet EVs during heat stress was first ascribed a role in the excessive activation of coagulation in a non‐human primate model of heatstroke (Bouchama et al., 2008). It seems unlikely that the increase in platelet EVs observed in the present analysis reflects this pathophysiological process, considering the moderate level of heat strain attained. Indeed, there is little evidence of altered haemostasis during moderate heat strain (Borgman et al., 2019). In contrast, moderate heat strain is generally associated with an increase in pro‐ and/or anti‐inflammatory cytokines (Faulkner et al., 2017; Hoekstra et al., 2018; Laing et al., 2008). It is possible that the increase in platelet EVs might contribute to and/or reflect this inflammatory response. Regardless of the potential functional significance of an increase in total and platelet EVs during heat stress, a new observation of the present analysis is that heat acclimation does not minimize this increase. Animal models have shown that heat adaptation upregulates cytoprotective pathways that lead to greater cellular resilience to various stressors (Murray et al., 2022). The cellular adaptations of human heat adaptation are less understood, although several studies have considered elements of the heat shock response (Amorim et al., 2015; Hoekstra et al., 2020). The present analysis adds to this body of knowledge by showing that short‐term passive heat acclimation does not alter basal concentrations of circulating EVs nor their change during moderate passive heat strain. The fact that heat acclimation did not minimize the increase in platelet EVs during heat stress might reflect previous studies showing that heat acclimation does not alter platelet function (Hue et al., 2004; Rama et al., 2016; Saat et al., 2005; Vesić et al., 2013) or inflammatory responses (Costello et al., 2018; Willmott et al., 2018; Yamada et al., 2007) during heat stress.

### 4.1 Limitations

When interpreting the present results, some limitations should be considered. First, no a priori sample size calculation was performed. It is possible that the analyses were underpowered to detect further statistical differences with heat stress and/or heat acclimation. However, effect sizes were generally small to medium (*d*_av_ *<* 0.8; Table 2) for changes that were not significant. Perhaps most important is whether the analyses were adequately powered to observe physiologically relevant changes in EV concentrations. To our knowledge, a physiologically meaningful change in EV concentration has not been determined for heat stress. Accordingly, the mean difference with 95% confidence interval (Table 2) might help to guide the interpretation of the results and/or power future studies. Second, the results are specific to healthy young adults. Future work could consider the effect of heat stress and/or heat acclimation on circulating EVs of individuals who present with elevated basal concentrations. Third, the results are specific to passive heat stress that induced moderate heat strain and to a 7‐day passive heat acclimation protocol. Future work could determine whether similar results are observed during severe heat strain and/or longer‐term heat acclimation. Fourth, blood samples were centrifuged once before storage, whereas two centrifugations are recommended to avoid residual cells within plasma that could be counted as artefactual EVs. This might have increased the absolute concentrations of EVs, although it is unclear whether it had an impact on the subsequent change during heat stress and/or heat acclimation. Lastly, the change in core temperature was greater post‐acclimation, in order to match absolute core temperature between pre‐ and post‐acclimation visits. It is unknown whether absolute core temperature or change in core temperature modulates EV concentrations during heat stress.

## 5 CONCLUSION

This exploratory analysis shows that moderate passive heat strain increases the circulating concentration of total and platelet EVs and that 7 days of passive heat acclimation does not alter this increase. The results also show that moderate heat strain does not alter circulating concentrations of endothelial, leucocyte or erythrocyte EVs. Future studies are needed to determine the functional implications, if any, of the increase in total and platelet EVs during heat stress.

## AUTHOR CONTRIBUTIONS

The experiments were performed within the Human Integrative Laboratory at Centre ÉPIC of the Montreal Heart Institute and in Dr Catherine Martel's laboratory at the Montreal Heart Institute. Nicholas Ravanelli, Catherine Martel and Daniel Gagnon contributed to the conception of the work. All authors contributed to the acquisition, analysis or interpretation of data for the work and to drafting of the work or revising it critically for important intellectual content. All authors approved the final version of the manuscript and agree to be accountable for all aspects of the work in ensuring that questions related to the accuracy or integrity of any part of the work are appropriately investigated and resolved. All persons designated as authors qualify for authorship, and all those who qualify for authorship are listed.

## CONFLICT OF INTEREST

None declared.

## Supporting information

Statistical Summary Document

## Data Availability

The original data from this work are available upon request to the corresponding author.
