# Supplementary material for: Impact of passive heat stress and passive heat acclimation on circulating extracellular vesicles: An exploratory analysis
Source: Exp Physiol. 2023 Jan 9;108(3):344–52. doi: 10.1113/EP090823 (PMC10103847; doi:10.1113/EP090823)
Supplement: Supplementary file 1 — Statistical Summary Document [file EPH-108-344-s001.docx]

**Manuscript Title:** Impact of passive heat stress and passive heat acclimation on circulating extracellular vesicles: an exploratory analysis.

**Authors:** Nicholas Ravanelli, Hadiatou Barry, Anthony R Bain, Laurent Vachon, Catherine Martel, Daniel Gagnon

**Animal model used, if applicable:** n/a

**Underlying hypothesis:** The objective of this study was to explore the effect of passive heat stress and subsequent heat acclimation on circulating concentrations of extracellular vesicles (EVs) in humans. This objective was achieved with a retrospective analysis. No *a priori* hypotheses were formulated.

**Definitions of ‘n’:** number of participants

**Statistical summary table:**

| Experimental question number* | Finding/ conclusion | Experimental location/ variable | Mean value | SD | n val. | P** | Units | Data comparisons | Statistical test | Any other variable  e.g. subjects’ age or sex | Figure/ table in which data are presented |
| --- | --- | --- | --- | --- | --- | --- | --- | --- | --- | --- | --- |
| How do the circulating concentrations of extracellular vesicles (EVs) change during passive when it is performed before and after heat acclimation | Passive heat stress increases the total concentration of circulating EVs and platelet-derived EVs. | CFSE^+^  EVs | Pre-acclimation  Baseline: 117060000  Heat: 145580000  Post-acclimation  Baseline: 133510000  Heat: 197290000 | 41530958  78085335  79512828  125055529 | 10 | 0.178  0.168 | EVs/mL | Heat vs. Baseline | 2-way repeated measured ANOVA with Fisher’s LSD post-hoc test | 4 females/6 males, 25 ± 4 years, 1.72 ± 0.08 m, 71.6 ± 9.0 kg, physical activity level: 156 ± 71 min/week | Figure 2 and Table 2 |
|  |  | CFSE^+^MHCI^+^  EVs | Pre-acclimation  Baseline: 18796000  Heat: 40599000  Post-acclimation  Baseline: 22269000  Heat: 58041000 | 10631303  24242717  15132624  52607568 |  | **0.011**  0.085 |  |  |  |  |  |
|  |  | CFSE^+^MHCI^+^CD41^+^  EVs | Pre-acclimation  Baseline: 18534000  Heat: 40367000  Post-acclimation  Baseline: 22022000  Heat: 56876000 | 10603920  24198880  15118631  51748743 |  | **0.010**  0.087 |  |  |  |  |  |
|  |  | CFSE^+^MHCI^+^CD62e^+^  EVs | Pre-acclimation  Baseline: 105120  Heat: 176370  Post-acclimation  Baseline: 105830  Heat: 223040 | 87277  293939  97576  271443 |  | 0.365  0.113 |  |  |  |  |  |
|  |  | CFSE^+^MHCI^+^CD45^+^  EVs | Pre-acclimation  Baseline: 12084  Heat: 13166  Post-acclimation  Baseline: 30462  Heat: 26553 | 9452  11550  46983  22732 |  | 0.823  0.732 |  |  |  |  |  |
|  |  | CFSE^+^CD235a^+^  EVs | Pre-acclimation  Baseline: 633690  Heat: 126710  Post-acclimation  Baseline: 119210  Heat: 240360 | 1361264  88254  67823  231291 |  | 0.270  0.084 |  |  |  |  |  |

*You may use multiple lines for the same question to indicate multiple comparisons

** Authors may wish to make the text bold where p is considered significant against a stated confidence limit.
